# Toxicities, intensive care management, and outcome of chimeric antigen receptor T cells in adults: an update

**DOI:** 10.1186/s13054-024-04851-0

**Published:** 2024-03-05

**Authors:** Mathieu Bellal, Jolan Malherbe, Gandhi Damaj, Damien Du Cheyron

**Affiliations:** 1 Department of Medical Intensive Care, Caen University Hospital, Avenue de la côte de nacre, 14000 Caen, France; 2 UNICAEN, INSERM UMRS U1237 PhIND, Normandie Univ, 14000 Caen, France; 3 Hematology Institute, Caen University Hospital, 14000 Caen, France

**Keywords:** Haematological malignancies, CAR-T cell therapy, Toxicities, Intensive care management

## Abstract

**Background:**

Chimeric antigen receptor T cells are a promising new immunotherapy for haematological malignancies. Six CAR-T cells products are currently available for adult patients with refractory or relapsed high-grade B cell malignancies, but they are associated with severe life-threatening toxicities and side effects that may require admission to ICU.

**Objective:**

The aim of this short pragmatic review is to synthesize for intensivists the knowledge on CAR-T cell therapy with emphasis on CAR-T cell-induced toxicities and ICU management of complications according to international recommendations, outcomes and future issues.

**Graphical abstract:**

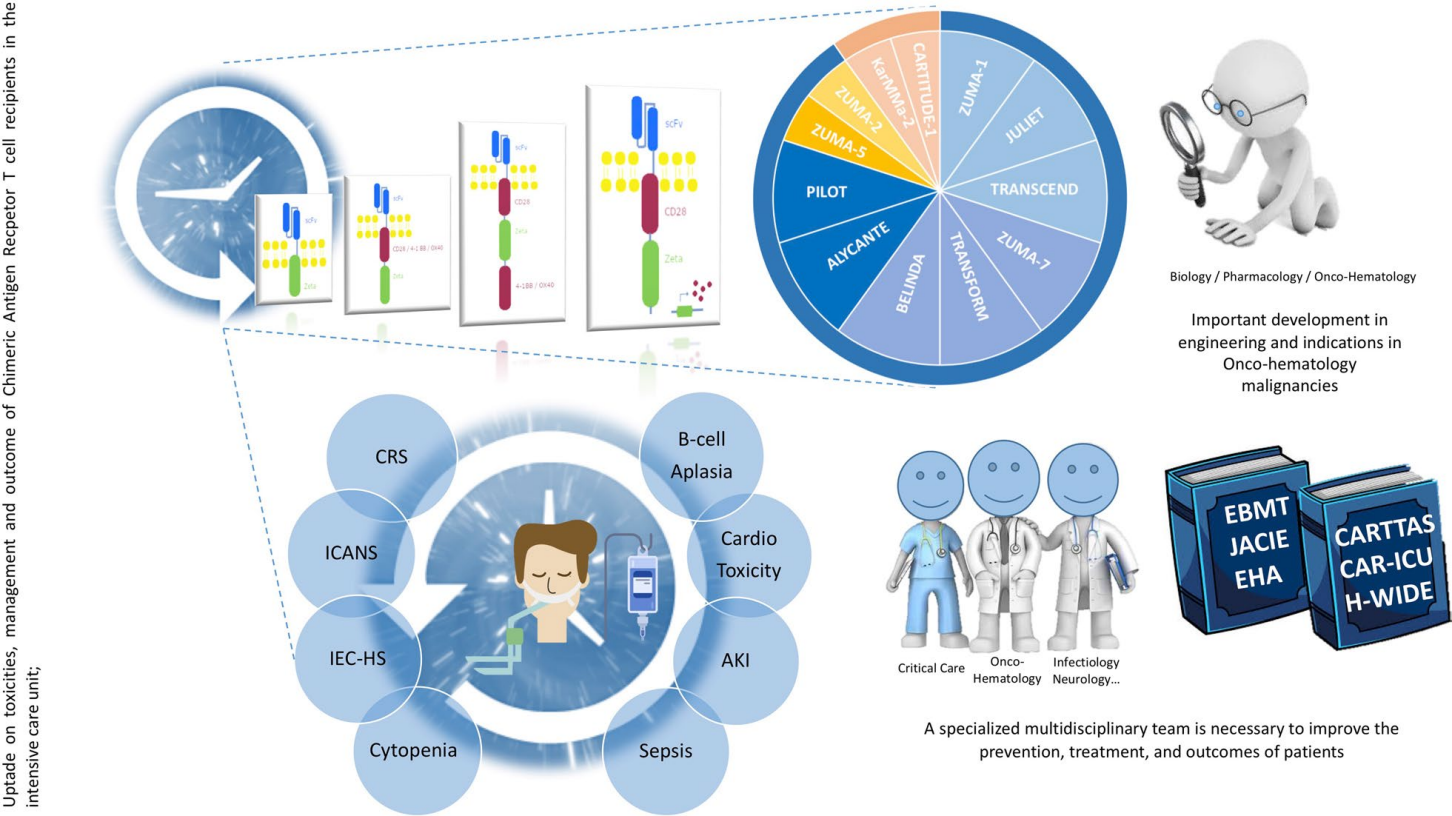

## Key points

- **Question**: What is the role of intensive care in the field of indications, toxicities management, and outcomes after CAR-T cell therapy.
- **Findings**: CAR-T cell therapies are developing rapidly and have an increasingly wide range of indications in haematological malignancies, as well as potential for treating solid cancers and autoimmune diseases in the near future. Despite improved survival rates, many patients present severe life-threatening toxicities that may require intensive care management, including cytokine release syndrome, immune effector cells associated neurotoxicity syndromes, immune effector cells associated haemophagocytic lymphohistiocytosis-like syndrome, infections, cardiovascular and renal specific toxicities.
- **Meaning**: This short pragmatic update reports the main toxicities after CAR-T cell therapy, the main retrospective observational studies of patients admitted to the ICU for early complications, and a summary of international recommendations for current practice in the medical intensive care unit.

## Mechanism of engineering and historic basing trials for CAR-T cell therapy

**Fig. 1:**
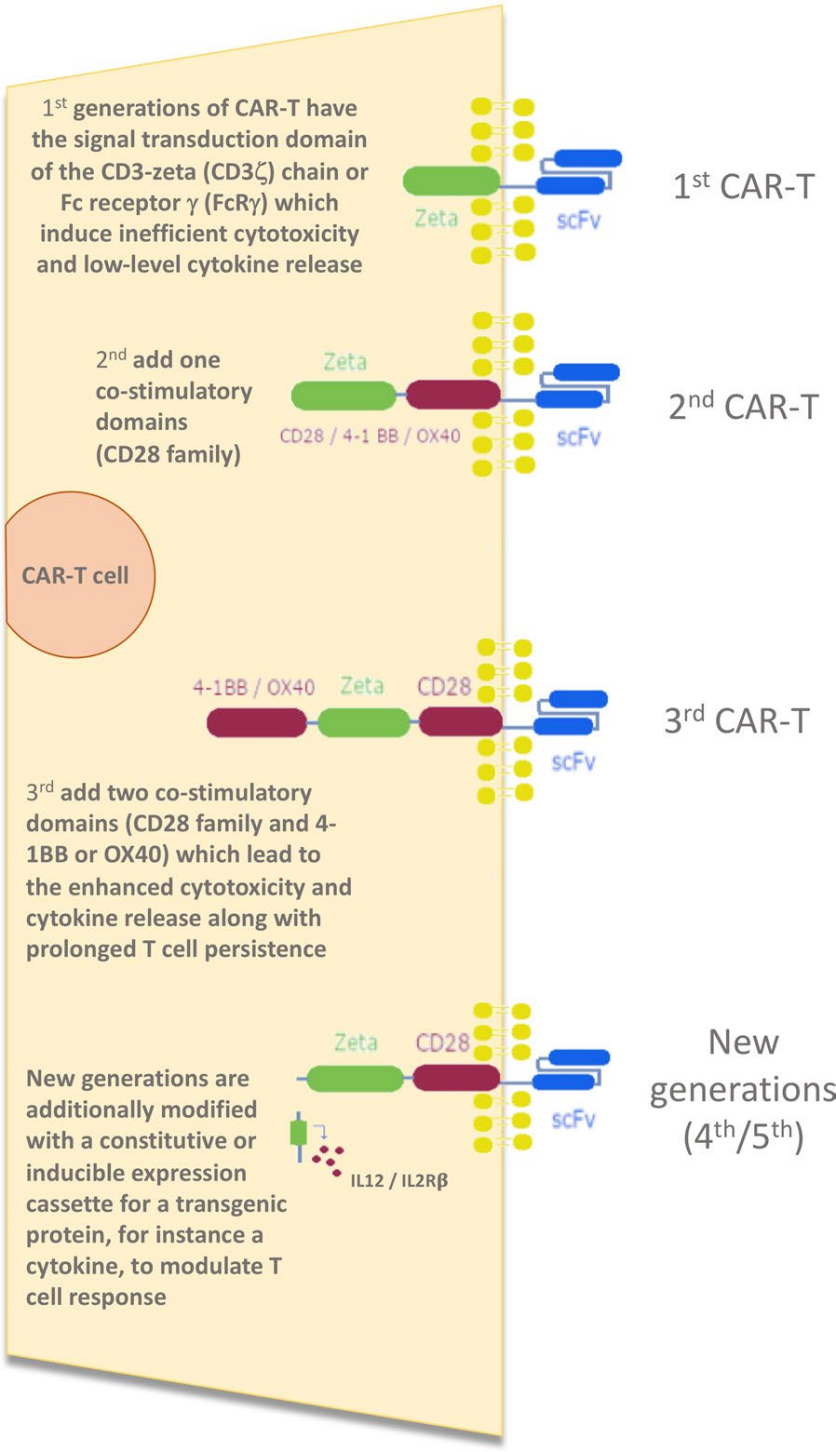
Five generation of CAR-T (adapted from [2, 3])

Chimeric antigen receptor T cell (CAR-T) immunotherapy is a new autologous cellular therapy that has been developed as an antitumor treatment. Its indications and the number of eligible patients have dramatically expanded over the past decade. Patient’s T cells from peripheral blood are engineered ex vivo with a recombinant T cell receptor (TCR) or a chimeric antigen receptor (CAR), which mediates antibody-targeted recognition and enhances T cell function upon binding [1]. CARs are synthetic receptors consisting of an antigen-binding domain-like extracellular single-chain variable fragment (scFv), transmembrane (TM), and an intracellular domain with tyrosine-based activation motifs (ITAMs) and co-stimulatory signal. The intracellular parts may be different and define five generations of CARs, which are summarized in Fig. 1 [2, 3].

**Fig. 2:**
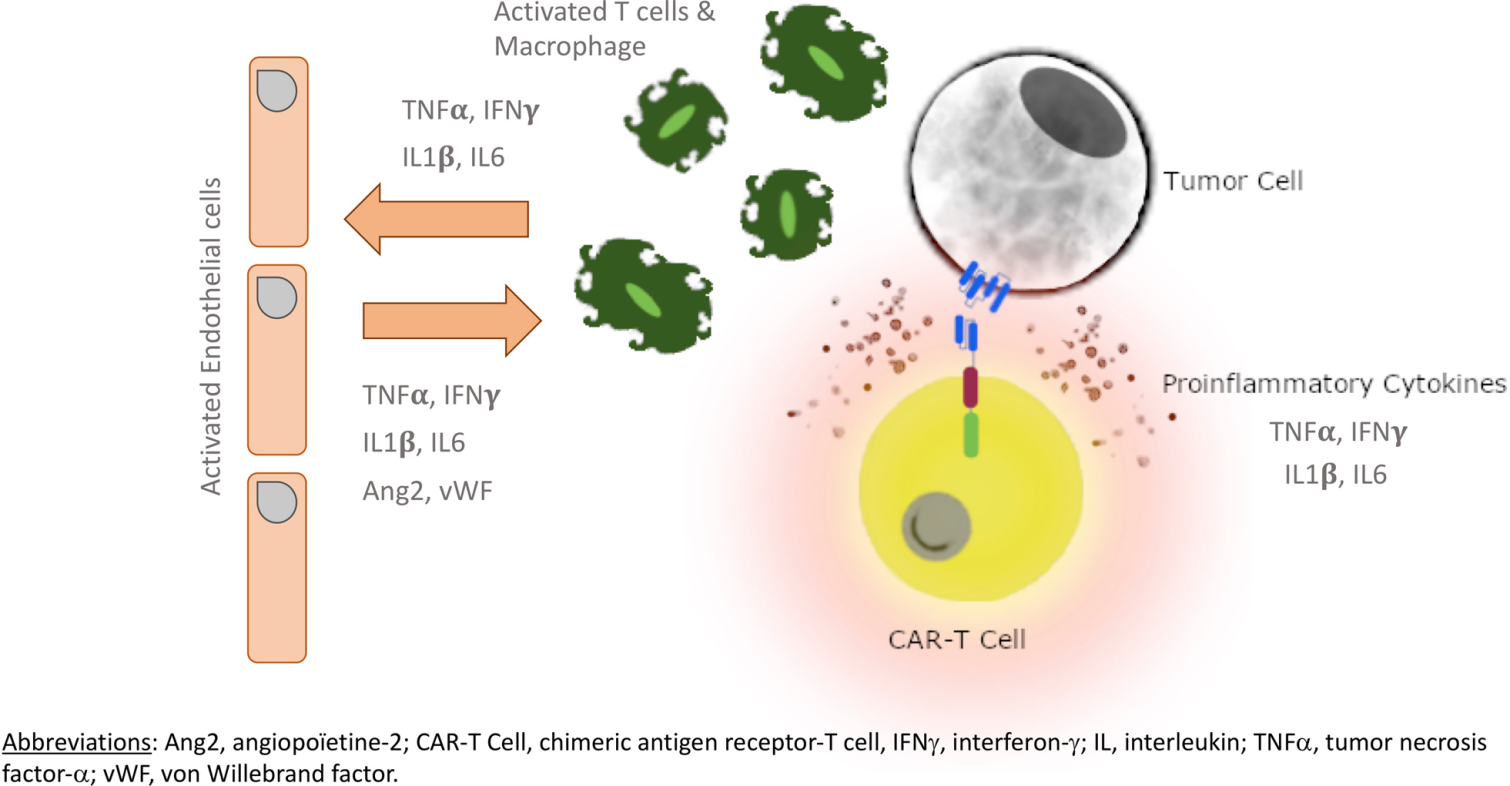
Antitumour mechanisms of CAR-T and cytokine release results in bystander activation of other immune cells (adapted from [7])

For B cell malignancies, CARs generally bind to CD19 targets and redirect the patient’s own cells to kill tumour cells in 3 main steps (Fig. 2): (1) the antigen-binding domain of CARs recognizes the CD19 antigen on the B cell; (2) the CD3ζ chain signalling domain induces T cell activation and secretion of cytokines; and (3) the co-stimulatory domains increase T cell activation and enhance the cytolytic function [4].

Based on phase 2 or 3 trials, anti-CD19 CAR-T cells have demonstrated efficacy in the treatment of paediatric and adult acute lymphoblastic leukaemia (ALL), adult refractory or relapsed high-grade B cell non-Hodgkin lymphoma (NHL) (for diffuse large B cell lymphoma (DLBCL), primary mediastinal B cell lymphoma and mantle cell lymphoma), indolent B cell NHL (for follicular lymphoma). Anti-BCMA (B cell maturation antigen) CAR-T cells have demonstrated efficacy in the treatment of multiple myeloma (MM) [5–22] (summarized in Table 1).

**Table 1:** Development of clinical trials of CAR-T cell therapy

	Methods of study and *Indication of treatment*	Study	Product	CAR-T infused/total number of patients	ORR/CR at day 90 (%)	Grade > 2 CRS/neurotoxicity (%)
r/r HBCL	Phase II *More than (* >*) 2 lines of chemotherapy*	JULIET [9]	Tisagenlecleucel (Kymriah™)	115/238	53/40	22/12
	Phase II > *2 lines*	ZUMA-1 [10]	Axicabtagene ciloleucel (Yescarta™)	101/111	83/58	13/31
	Phase II > *2 lines*	TRANSCEND [11]	Lisocabtagene maraleucel (Breyanzi™)	269/344	73/53	2/10
	Phase III > *1 line with HSCT*	BELINDA [12]	Tisagenlecleucel (Kymriah™)	155/322	46/28	5/2
	Phase III > *1 line with HSCT*	ZUMA-7 [13]	Axicabtagene ciloleucel (Yescarta™)	170/359	83/65	6/21
	Phase III > *1 line with HSCT*	TRANSFORM [14]	Lisocabtagene maraleucel (Breyanzi™)	184/232	86/66	1/4
	Phase II > *1 line without HSCT*	ALYCANTE [15]	Axicabtagene ciloleucel (Yescarta™)	62/69	69/66	8/14
	Phase II > *1 line without HSCT*	PILOT [16]	Lisocabtagene maraleucel (Breyanzi™)	61/74	80/54	1,5/5
r/r MCL	Phase II > *2 lines*	ZUMA-2 [17]	Brexucabtagene autoleucel (Tecartus™)	68/74	91/68	15/31
r/r IBCL	Phase II > *2 lines*	ELARA [18]	Tisagenlecleucel (Kymriah™)	97/119	86/68	0/3
	Phase II > *2 lines*	ZUMA-5 [19]	Axicabtagene ciloleucel (Yescarta™)	148/153	94/77	7/19
r/r B-ALL	Phase II	ZUMA-3 [20]	Brexucabtagene autoleucel (Tecartus™)	55/71	71/56	24/25
r/r MM	Phase II > *2 lines*	CARTITUDE-1 [21]	Ciltacabtagene autoleucel (Carvykti™)	97/113	98/82	4/2
	Phase III > *2 lines*	KarMMa-3 [23]	Idecabtagene Vicleucel (Abecma™)	225/386	71/39	5/3

*r/r* relapsed/refractory, *HBCL* high-grade B cell lymphoma, *MCL* mantle cell lymphoma, *IBCL* indolent B cell lymphoma, *B-ALL* B cell acute lymphoblastic leukaemia, *MM* multiple myeloma, *HSCT* haematopoietic stem cell transplantation, *ORR* overall response rate, *CR* complete response rate, *CRS* cytokine release syndrome

Currently, six commercially products have been approved by the Food and Drug Administration (FDA) for adult patients: Two autologous anti-BCMA CAR-T cell products (idecabtagene vicleucel and ciltacabtagene autoleucel) and four autologous second-generation anti-CD19 CAR-T cell products (tisagenlecleucel, lisocabtagene maraleucel, axicabtagene ciloleucel, and brexucabtagene autoleucel). They differ in the co-stimulatory domain (4-1BB for tisagenlecleucel and lisocabtagene maraleucel and CD28 for axicabtagene ciloleucel and brexucabtagene autoleucel) and by the transduction vector (lentivirus for Tisagenlecleucel and Lisocabtagene maraleucel and retrovirus for Axicabtagene ciloleucel and Brexucabtagene autoleucel). Thus, the expansion speed and duration of action differ between products, ranging from weeks for axicabtagene to months for tisagenlecleucel [23].

The whole process of treatment with anti-CD19 CAR-T cells includes patient’s selection, determining eligibility, leukocyte apheresis, and bridging therapy to stabilize the disease and prevent rapid progression during the 3–8 weeks of the cell manufacturing process, which is the *vein-to-vein time* between leukapheresis and infusion. This is followed by lymphodepletion conditioning and CAR-T cell infusion, after which complications may occur [24].

This short pragmatic review for intensivists focuses on short-term (admission to day 28) and medium-term (day 29–100) complications, including severe life-threatening toxicities possibly requiring admission to intensive care unit (ICU). Management methods for these complications were developed based on the current literature and recent recommendations derived from a comprehensive review on the topic from the European Society for Blood and Marrow Transplantation (EBMT), Joint Accreditation Committee ISCT-Europe (JACIE), and European Haematology Association (EHA) [25].

## Short-term complications

### Tumour lysis syndrome (TLS)

TLS has been reported in 5–17% of CAR-T recipients [26] and is characterized by hypocalcaemia, hyperkalaemia, metabolic acidosis, hyperphosphatemia, hyperuricemia, and renal failure. TLS should be prevented and managed with adequate monitoring and standard care, including control of potassium and phosphorus intake during the risk period, hyperhydration, and reducing the level of uric acid (with allopurinol or rasburicase). Despite optimal care, severe acute kidney injury (AKI) remains a frequent complication of TLS [27] and may require dialysis according to the AKI guidelines [28].

### Infections and sepsis

CAR-T cell recipients have high risk of sepsis, which is one of the main reasons for ICU admission. A high proportion of patients who receive CAR-T cell therapy develop typical bacterial (20%), viral (5–10%), and fungal (< 5%) infections within the first 28 days after infusion [29]. Most of these infections (80%) occur within the first 10 days, and most patients present with lower-respiratory tract infections.

Risk factors for infection after CAR-T infusion include neutropenia, previous antitumor treatment regimens, the CAR-T cell dose, high grade of cytokine release syndrome (CRS) or immune effector cells associated neurotoxicity syndromes (ICANS), and their treatments. Because long-lived plasma cells do not express CD19, so humoral immunity to viruses is preserved, and the occurrence of severe viral infections remains rare with anti-CD19 CAR-T [30]. To date, few studies have specifically addressed the issue of viral infections or reactivations in patients receiving anti-BCMA CAR therapy. However, Wang et al. recently reported that viral infections or reactivations due to double-stranded DNA viruses like herpes virus, adenovirus, and BK or JC viruses were common adverse events in patients receiving anti-BCMA [31].

There are no standardized approaches to antimicrobial prophylaxis regimens for CAR-T cell recipients. Fever after lymphodepletion and CAR-T infusion requires, however, prompt empiric antimicrobial therapy, because infections and sepsis are an important determinant of increased morbidity and mortality [32].

### Cytokine release syndrome (CRS)

CRS is the most common acute toxicity induced by CAR-T cell therapy. It is characterized by systemic inflammatory reaction (a “*cytokine storm”*) with flu-like symptoms, hypoxemia, and haemodynamic instability. It is staged into 4 grades according to consensus criteria of the American Society for Transplantation and Cellular Therapy (ASTCT) [33]. Pathophysiologically, CRS leads to the release of effector cytokines which activate the monocyte/macrophage system and induce the production of pro-inflammatory chemokines. In preclinical models, the main cytokine with the highest concentration is IL-6, which explains the first-line use of the anti-IL-6 receptor tocilizumab for CRS.

In a recent review, the incidence of CRS grade > 2 was reported in 29% of treatments of ALL and 20% of treatments of refractory or relapsed high-grade B cell NHL [34]. Overall, grade > 2 is reported in 10–30% of cases and appears within the first 14 days after CAR-T infusion due to CAR-T activation [35]. Risk factors of CRS grade > 2 include tumour burden, active infection, baseline inflammation, the CAR-T dose and product, and the intensity of lymphodepletion conditioning.

**Fig. 3:**
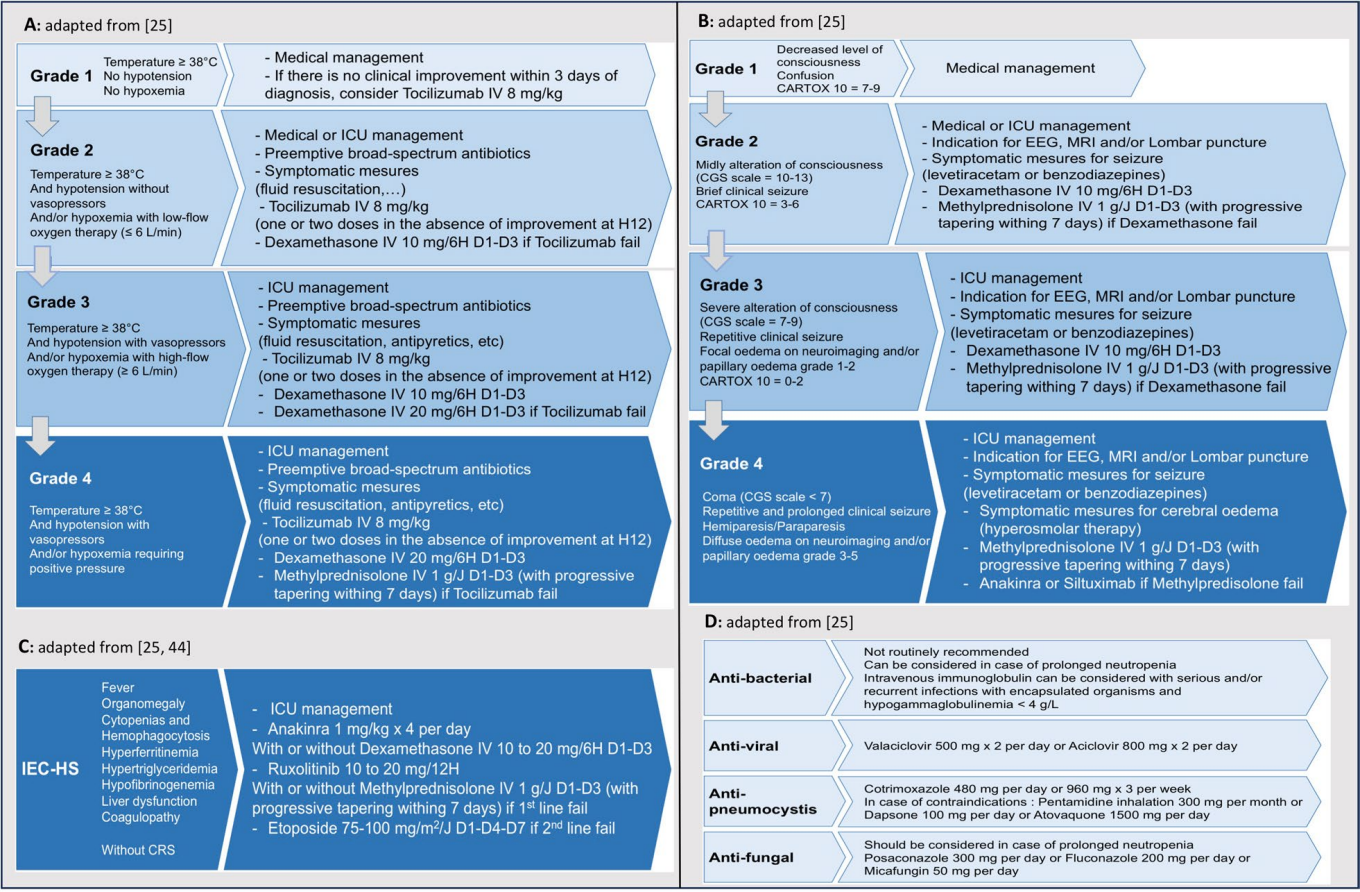
Algorithm of management of CRS (**A**), ICANS (**B**), IEC-HS (**C**), and infections (**D**)

CRS management involves standard of care for haemodynamic instability and hypoxemia, and empiric and broad-spectrum antibiotics based on institutional protocols similar to those used in neutropenic patients with sepsis. Tocilizumab is recommended as the first-line treatment for isolated CRS after CAR-T treatment (from grade 1, if there is no clinical improvement within 3 days of diagnosis and no other differential diagnosis, to grade 4) [25, 36, 37]. If tocilizumab fails to control CRS after two doses, corticosteroids like IV dexamethasone should be administered (summarized in Fig. 3A) [25]. If tocilizumab and corticosteroids fail to control CRS, siltuximab (IL-6 antagonist) or anakinra (IL-1 receptor antagonist) could be considered, but limited clinical data are available for isolated CRS, in contrast to CRS associated with ICANS [38].

### Immune effector cells associated neurotoxicity syndrome (ICANS)

ICANS is the second most common adverse event of CAR-T cell therapy and can occur with or without CRS or after it. It is characterized by tremor and myoclonus, alterations in mental status, dysarthria or aphasia, deterioration in handwriting, or seizures [39]. It is staged into 4 grades by the ASTCT consensus criteria [33]. ICANS grade > 2 is reported in 12–30% of cases and appears between 3 and 5 days after CAR-T infusion. Pathophysiology includes endothelial activation, collapse of the blood–brain barrier, migration of immune cells into the central nervous system, and release of pro-inflammatory cytokines following CAR-T cell activation.

Only 10% of patients develop delayed ICANS, with a time-interval greater than 3 weeks after CAR-T infusion [40]. Diagnostic work-up should include cerebral computed tomography scan or magnetic resonance imaging (MRI), electroencephalography, and lumbar puncture. ICANS grading provides an overall assessment of neurological function by integrating the 10-point Immune Effector Cell Encephalopathy (ICE) score (previously CAR-T cell therapy-associated Toxicity (CARTOX)): from ICANS grade 0 if ICE score is 10 points to ICANS grade 4 if ICE score is 0 point, with 4 points for orientation, 3 points for naming objects, one point for “knowing how to follow an order”, or “writing a sentence”, or “counting backwards” [41].

Management of ICANS includes symptomatical treatment for seizures and status epilepticus, followed by corticosteroids in cases of ICANS grade > 1. If necessary, neuroprotective treatment should be considered in identified severe cerebral oedema (grade 4). After one to three days at the full dose, it is recommended that a gradual taper of steroids begins as soon as symptoms are controlled and disappear (without a clearly established dose and tapering duration) [25]. In animal models, tocilizumab failed to prevent delayed lethal toxicity, but anakinra did not [36]. These preclinical findings have led to recommendations for the second-line use of anakinra in ICANS after failure of corticosteroid therapy, followed by siltuximab in ICANS refractory to anakinra (severe grade 4), although limited clinical data are available (summarized in Fig. 3B) [25].

### Immune effector cell—associated haemophagocytic lymphohistiocytosis-like syndrome (IEC-HS)

IEC-HS results from mononuclear phagocytic system activation, a dysregulated immune response, and a severe cytokine storm specifically following CAR-T cells with a clinical independence of CRS. It is characterized by fever, organomegaly, cytopenias by haemophagocytosis in bone marrow, liver dysfunction, dysfibrinogenemia, hyperferritinemia and hypertriglyceridemia. IEC-HS diagnosis is usually based on the H-score, Anderson criteria and haemophagocytic lymphohistiocytosis syndrome (HLH)-2004 criteria in paediatric population [42, 43].

The new expert consensus and consensual diagnostic criteria to recognized IEC-HS following CAR-T cell therapy allow earlier clinical recognition in order to initiate appropriate therapeutic management as quickly as possible [44].

IEC-HS management involves anakinra with or without corticosteroids. There are also concerns regarding potential adverse effects of corticosteroid therapy on CAR-T cell function and persistence, but most data suggest that short-term corticosteroids can be employed in the treatment of complications (including CRS/ICANS/IEC-HS) without a clear increase in the relapse rates of malignancies [45]. Ruxolitinib (a JAK/STAT pathway inhibitor with promising preclinical results) is a second-line option based on its use in hematopoietic cell transplantation [46, 47]. Chemotherapy drug such as etoposide might also be used in refractory IEC-HS, although there is a high risk of altering the efficacy of CAR-T. In cases of associated neurotoxicity, lumbar puncture with cytarabine or steroid infusion can be considered [48], but limited clinical data are available (summarized in Fig. 3C).

Because of overlapping features and pathological similarities, HLH-like manifestations are frequently seen in patients with severe CRS/ICANS, infections or progressive malignancy but are not defined as IEC-HS, with a rate ranging from 1 to 33% [42, 49]. Their management is based on etiological treatment and systemic corticosteroids with or without etoposide combination therapy [43].

### Cardiovascular toxicity

Cardiovascular complications are reported in 10–20% of CAR-T cell recipients. Risk factors include CRS grade > 1, disease burden, pre-existing cardiac dysfunction, and exposure to cardiotoxin therapy, such as anthracyclines or tyrosine kinase inhibitors [50]. Currently, there are no formal guidelines for risk stratification. Nevertheless, in a recent review, Gutierrez et al. reported a group of patients with high risk for cardiac comorbidities before CAR-T cell infusion including prior or current cardiomyopathy, heart failure with reduced left ventricular ejection fraction (< 50%), prior history of myocardial infarction or coronary revascularization, significant valve disease, and age > 65 years [51].

The mechanisms involved in cardiovascular dysfunction are thought to be primarily mediated by the systemic inflammation of CRS, particularly IL-6. In a recent trial, CAR-T-related severe cardiovascular events were independently associated with increased non-relapse mortality and overall mortality risk [52]. ICU management is not specific, but cardiac MRI has emerged as an interesting tool for the diagnosis of CAR-T cell-related cardiotoxicity and differential diagnoses [53, 54]. Because of the close interaction between CRS and CAR-T cell-related cardiotoxicity, cardiovascular complications must be managed with intravenous tocilizumab and are associated with rapid improvement [52]*.*

### Kidney toxicity

AKI is frequent after CAR-T cell therapy, with an estimated incidence of 18.6% [55, 56]. Several mechanisms can explain AKI after CAR-T cell infusion, such as vasodilatory shock after CRS, sepsis, immunoallergic tubulointerstitial nephritis, and TLS. Like any patients with haematological malignancies, AKI and dialysis are strongly associated with increased mortality [57]. Nevertheless, there is no specific management for AKI after CAR-T cell therapy, and dialysis modalities may rely on AKI guidelines [28].

## Medium-term complications

### Delayed TLS, CRS, and ICANS

All the major short-term syndromes described thus far may occur later and should be managed in the same way.

### B cell aplasia and hypogammaglobulinemia

Most of the antigens targeted by CAR-T cell therapy are not exclusively specific to tumours but are also expressed by non-malignant tissues (*off-tumour* and *on-target* toxicity). Anti-CD19 or anti-BCMA CAR-T cells target B cell CD19 or BCMA antigens, respectively, so patients can develop B cell aplasia and profound hypogammaglobulinemia. These adverse effects were reported in 25% of cases at 12 months in the ZUMA-1 trial and associated with sino-pulmonary infections [10]. Intravenous immunoglobulins (0.4 g/kg/month) or subcutaneous immunoglobulins (0.1 g/kg/week) are the standard treatment for hypogammaglobulinemia below 4 g/L associated with recurrent infections. Discontinuation of immunoglobulin administration should be guided by the recovery of functional B cells. Notably, 65% of patients receiving CAR-T cell recovered a normal level of absolute B cell numbers with a median time of 12 months (range 2–59 months) [58].

### Delayed and prolonged cytopenias

**Fig. 4:**
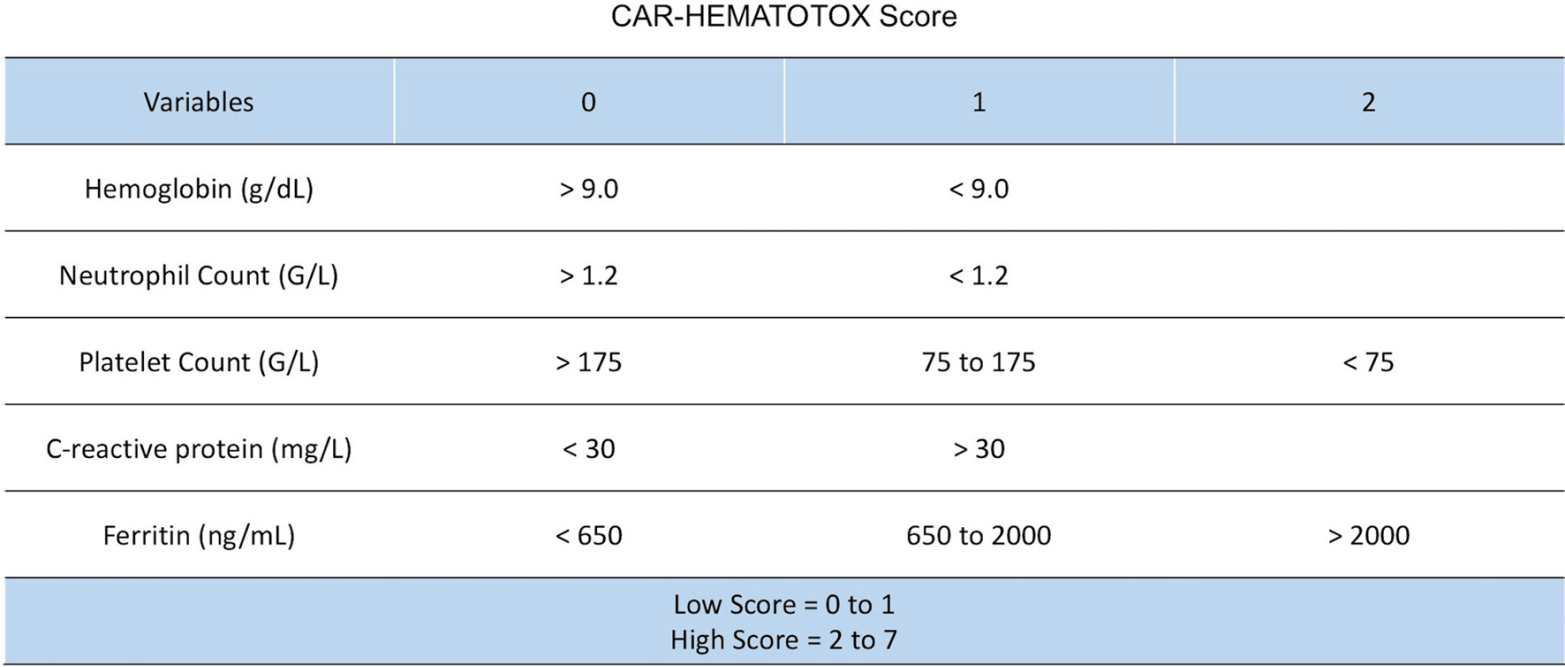
CAR-HEMATOTOX: to be determined before lymphodepletion to discriminate between a low and a high risk for haematotoxicity, from [60]

Delayed haematological toxicity may affect up to 65% and increases morbidity and mortality after CAR-T treatment. Several mechanisms can explain prolonged and late cytopenia, such as hyperinflammatory syndrome like IEC-HS, immune-mediated hematopoietic stem cell suppression, mature blood cell destruction, transplant-associated thrombotic microangiopathy, primary disease relapse, and secondary marrow neoplasm. Clinical trials have reported a high incidence of persistent grade > 2 neutropenia (30–40%), thrombocytopenia (20–30%), and anaemia (10–15%) after day 28. In these cases, bone marrow biopsy may be useful to exclude recurrent disease, secondary or non-specific HLH or secondary myelodysplasia [59]. The CAR-HEMATOTOX model is an easy-to-use risk stratification tool of delayed haematological toxicity that was evaluated in 258 patients with refractory or relapsed DLBCL receiving axicabtagene ciloleucel or tisagenlecleucel [60]. This score includes markers associated with the patient’s hematopoietic reserve and systemic inflammatory status prior to lymphodepletion conditioning and injection of CAR-T cells without being predictive of the occurrence of CRS/ICANS/IEC-HS (summarized in Fig. 4). It is associated with a risk of profound and prolonged cytopenias, infectious complications, prolonged hospitalization, and worse clinical outcomes (negative prognostic impact on overall response rate, progression-free survival and overall survival). A score between 2 and 7 is considered high and may indicate antimicrobial prophylaxis in cases of risk factors for sepsis, although there are no strong recommendations.

Platelet and packed red blood cell transfusion support may be necessary, and granulocyte colony-stimulating factor (G-CSF) can be used for severe neutropenia (< 0.5 G/L). In addition, erythropoietin, thrombopoietin agonists, and IEC-HS directed therapy may have a role in these severe situations. Finally, if stem cells from a prior autologous or allogeneic bone marrow transplantation have been persevered and are available for use, a stem cell boost can be used as a last resort in cases of refractory cytopenias [61].

### Infections and antimicrobial prophylaxis

In a recent retrospective analysis from the DESCAR-T registry, Lemoine et al. reported the occurrence of late non-relapse mortality after CAR-T cell therapy for DLBCL. In a median follow-up of 12.4 months, most of them were due to infections (52%) [62]. While post-opportunistic infections are bacterial in the first 30 days, viral infections predominate beyond day 30, which mainly occur in the upper- and lower-respiratory tracts. Late reactivation of herpes virus has been reported. Consequently, antimicrobial prophylaxis is warranted until immune reconstitution (summarized in Fig. 3D) [25]. Early and late post-CAR-T fungal infections appear to be rare. In a recent cohort study including 84 patients admitted to the ICU, only 3 (3.6%) developed fungal infections [63].

## Characteristics and outcome of patients admitted in the intensive care unit

A few years ago, through the CAR-ICU initiative, a task force of experts in CAR-T cell therapy has launched a practice survey in 11 US hospitals concerning practices for the management of side effects in CAR-T recipients [64]. They recorded CAR products, toxicities, targeted treatment, management practices and interventions in the ICU. The authors highlighted differences between centres in severity criteria on ICU admission for CRS, but not for ICANS. The management of complications in CAR-T patients was relatively consistent between centres, including the use of vasopressors, monitoring of neurotoxicity by electroencephalogram, prophylactic use of antiepileptic drugs and use of tocilizumab. Conversely, other therapies differed between centres, which included fluid resuscitation, mechanical ventilation requirement, and use of corticosteroids. The authors concluded that future studies were needed to homogenize practices and improve the prognosis of patients.

Recent studies have described the epidemiology, treatments, and outcome of multicentre cohorts of adult patients admitted to the ICU for short-term complications induced by CAR-T cell therapy [65–67]. Table 2 provides the main characteristics of CAR-T recipients in these observational cohort studies in ICU settings. In summary, the average age of patients admitted to the ICU after CAR-T varied from 57 to 60 years, with a majority being men (from 59 to 66%) and a maximum median SOFA score ranging from 4 to 5. The main indication for CAR-T cell therapy was DLBCL, followed by MM and ALL, which could be refractory or relapsed after 3 to 4 lines of standard chemotherapy. In the CAR-ICU [65] and CARTTAS [66] studies, the number of CAR-T recipients requiring transfer to the ICU for severe toxicity after CAR-T cell treatment ranged from 27 to 35%. In all studies, CRS occurred in around 70% of patients, with the proportion of severe CRS (grade > 2) ranging from 18 to 35% and occurring between 2 and 5 days after CAR-T cell infusion. ICANS occurred in 37 to 75% of patients, with the proportion of severe ICANS (grade > 2) ranging from 15 to 65% and occurring 1–6 days after CAR-T cell infusion. Furthermore, 22–30% of patients had a documented infection, but in the cohort examined by Valade et al., 98% of patients received broad-spectrum antibiotics in the context of neutropenia [67]. Of note, the rate of TLS was not reported, nor were those of cardiomyopathy and secondary HLH, except in the CAR-ICU study where the proportions observed were below 4%. Regarding artificial organ support therapies, almost a third of patients received vasopressors, and around 10% required mechanical ventilation. Less than 5% of CAR-T recipients required renal replacement therapy. Tocilizumab and corticosteroid drugs were used in 60–75% of cases as a first line of treatment in CAR-T-induced CRS, as recommended by the EBMT, JACIE, and EHA [25]. Finally, ICU and hospital mortality varied from 1.5 to 9% and from 12 to 17.5%, respectively.

**Table 2:** Characteristics of CAR-T recipients in ICU studies

Characteristics and variables	CAR-ICU study [65]	CARTTAS study [66]	Hospital-wide study [67]
Inclusion period	November 2017–May 2019	February 2018–February 2020	July 2017–December 2020
Type of study	Retrospective, cohort, multicentre study	Retrospective, cohort, multicentre international study	Retrospective, cohort, monocentre study
Nature of B cell malignancies	DLBCL, FL	DLBCL, FL, MM, ALL	DLBCL, ALL, MM
Nature of CAR-T cell therapy	Axicabtagene ciloleucel, Tisagenlecleucel	*NA*	Autologous CAR-T cells, Axicabtagene ciloleucel, Tisagenlecleucel, Brexucabtagene autoleucel, bb2121, Allogenic CAR-T cells (UCART19)
Lines of chemotherapy prior to CAR, median (range)	4 [1–11]	3 [2–4]	3 [3, 4]
Number of patients treated by CAR-T cell therapy	345	942	*NA*
Number of patients admitted to ICU, n (%)	120 (34.8)	258 (27.4)	71
Age, year, mean (SD) or median [range]	57 (15)	58 [43–66]	60 [37–67.5]
Sex (male), n (%)	79 (65.7)	144 (60)	42 (59)
Maximum SOFA score, median [range]	5 [1–21]	4 [2–7]	4 [2–6]
Number of patients with TLS, *n* (%)	*NA*	*NA*	*NA*
Number of patients with CRS, *n* (%)	81 (67.5)	200 (77.5)	33 (46)
Grade 3–4 CRS, *n* (%)	28 (34.6)	50 (19.4)	6 (18.1)
Time from infusion to maximum CRS, day (range)	5 [0–42]	NA	2 [1–3]
Number of patients with ICANS, *n* (%)	89 (74.2)	108 (41.5)	26 (37)
Grade 3–4 ICANS, *n* (%)	67 (75.3)	38 (14.7)	8 (30)
Time from infusion to maximum ICANS, day (range)	6 [2–74]	*NA*	1 [0–1]
Documented infections, *n* (%)	26 (21.6)	78 (30.2)	21 (30)
Documented cardiomyopathy, *n* (%)	3 (2.5)	*NA*	*NA*
Documented sHLH, *n* (%)	4 (3.3)	*NA*	*NA*
Documented acute renal failure, *n* (%)	8 (6.7)	*NA*	*NA*
Organ support			
Vasopressors, *n* (%)	22 (18.3)	74 (28.7)	20 (28)
High-flow oxygen therapy, *n* (%)	11 (9.2)	14 (5.4)	*NA*
Non-invasive ventilation, *n* (%)	2 (1.7)	0 (0)	4 (6)
Invasive mechanical ventilation, *n* (%)	14 (11.7)	26 (10)	
Renal replacement therapy, *n* (%)	3 (2.5)	12 (4.6)	1 (1.5)
Treatment of toxicities			
Tocilizumab, *n* (%)	87 (72.5)	166 (64.3)	49 (69)
Corticosteroid, *n* (%)	93 (77.5)	155 (60)	40 (56)
Siltuximab treatment, *n* (%)	9 (7.5)	*NA*	9 (12.6)
Anakinra treatment, *n* (%)	9 (7.5)	*NA*	2 (2.8)
Outcome			
ICU length of stay, day (range)	4 [1–22]	4 [1–10]	*NA*
ICU mortality, *n* (%)	11 (9)	14 (5.4)	1 (1.5)
Hospital length of stay, day (range)	24 [5–180]	16 [9–34]	*NA*
Hospital mortality, *n* (%)	21 (17.5)	36 (14)	8 (12)

*DLBCL* diffuse large B cell lymphoma, *FL* follicular lymphoma, *MM* multiple myeloma, *ALL* acute lymphoblastic leukaemia, *CAR-T* chimeric antigen receptor T cell, *SOFA* Sepsis-related Organ Failure Assessment, *CRS* cytokine release syndrome, *ICANS* Immune effector Cells Associated Neurotoxicity Syndrome, *sHLH* Secondary Haemophagocytic Lymphohistiocytosis Syndrome, *TLS* Tumour Lysis Syndrome, *NA* not applicable

Results are expressed as number (percentage), mean (standard deviation) or median [range]

Thus, these three retrospective observational studies of patients admitted to the ICU for early complications secondary to CAR-T cell administration show that the population is predominantly male and middle-aged population with an intermediate severity score (SOFA) for acute illness on admission to the ICU, with mainly haemodynamic failure and a relatively low mortality rate.

In the CAR-ICU trial, higher cumulative corticosteroid doses were associated with decreased survival rate, while CRS and ICANS toxicity grades or organ support did not impact the overall survival [64]. In the CARTTAS trial, frailty, bacterial infection, and lifesaving therapy within 24 h of ICU admission were identified as independent risk factors of 90-day mortality [66]. Similarly, Valade et al. identified reason for ICU admission (disease progression *vs* sepsis or CRS), performance status, and SOFA score as determinants of mortality [67]. Finally, mortality appears to be associated with the severity of the acute illness, particularly in patients whose performance status is impaired or whose malignant haematological disease is progressing. Altogether, multidisciplinary management of severe patients requires early recognition of life-threatening toxicity symptoms related to CAR-T cell therapies, rapid and maximal treatment of organ failures and infections, as well as perfect knowledge of the treatments specific to the management of CRS or ICANS, according to the evidence-based medicine and the international recommendations [25, 68]. Carefully selecting eligible patients and developing individualized patient management plans are required to improve the prognosis of these serious patients in the era of this new cell therapy with increasingly broad indications.

## Challenges and future issues

### New perspectives and improvement of CAR-T cell therapy

New CAR-T cell-based therapies continue to be developed and could prove beneficial for other B cell neoplasias. For example, anti-CD30 CAR-T cell recently demonstrated efficiency in refractory or relapsed Hodgkin Lymphoma, without neurologic toxicity [69].

Studies have suggested that upon target engagement, CAR-T cell therapy rapidly increases activation markers, including programmed cell death-1 (PD-1). The expression of PDL-1 on tumour cells associated with PD-1 activation on CAR-T led to the hypothesis that blocking this signalling cascade could increase the activation, proliferation, and cytolytic activity of CAR-T cell therapy [70, 71]. Thus, the combination of new immunotherapies may be able to improve treatment efficacy. For example, the phase 1/2 ZUMA-6 trial was designed to assess the value of treating refractory or relapsed high-grade DLBCL with a combination of CAR-T cell therapy and monoclonal antibody targeting PDL-1 [72].

The use of allogeneic CAR-T cells from living donors is another approach that could change the therapeutic landscape of CAR-T cell therapy. The potential expected benefits are the possible standardization of CAR-T cell products, the possibilities of multiple cell modifications and using an industrialized process to reduce cost, and the immediate availability of these cryopreserved products for patient treatment. In this respect, the phase 1 ALPHA trial was designed to evaluate the benefit of allogeneic CAR-T cell therapy (ALLO-501 and ALLO-647™) in the treatment of refractory or relapsed high-grade DLBCL or follicular lymphoma [73]. Similarly, Mailankody et al. reported the feasibility and safety of allogeneic anti-BCMA CAR-T cell therapy for refractory or relapsed MM [74].

Resistance or relapse after CAR-T cell therapy can be explained by a mechanism of target repression (i.e. loss of CD19 expression). Thus, the creation of autologous CAR-T cells targeting two antigenic profiles, CD19 and CD22, represents an innovative approach to counteract the acquisition of tumour cell resistance to CAR-T through loss of the mono-antigenic target. The efficacy of bispecific CAR-T cell has been recently tested in patients with refractory or relapsed ALL [75], and with refractory or relapsed high-grade DLBCL [76].

Finally, studies using new CAR-T cell strategies are underway for several haematological malignancies to challenge the monopoly of commercial autologous CAR-T. Their main objectives are to improve response rates, avoid the acquisition of resistance, minimize adverse effects, and reduce manufacturing time (a recent trial have used YTB323 or rapcabtagene autoleucel, an autologous CD19-directed CAR-T cell generated by an innovative platform that produces CAR-T in 2 days [77]).

### Future strategies to limit toxicities and improve prognosis

Because high rates of complications have been reported in numerous trials, ranging from 40 to 90% for all grades CRS and from 20 to 65% for ICANS [25], several phase 1/2 studies are warranted to assess new prophylactic or curative treatment strategies, particularly by using granulocyte–macrophage colony-stimulating factor (GM-CSF) or anti-IL-1-R. Current research is also focusing on sparing corticosteroid therapy, which may ultimately be responsible for reduced survival by modifying the engineering of CAR-T cell therapy [78], as well as better haemopathy control prior to treatment [79]. Finally, novel cell products like CAR-natural killer cells or CAR-macrophages may have several benefits over CAR-T cells, without surge of inflammatory cytokines, while lowering the risk of CRS and ICANS and reducing risk of “*on-target/off-tumour*” toxicity [80].

Although a better understanding of pathophysiology has improved the quality of patient care in the ICU setting and has led to increased in-hospital and overall survival [63, 81], CAR-T cell management is currently based on few recommendations with high levels of evidence. All this could evolve over the coming years as the indications for this immunotherapy are extended to autoimmune diseases and solid cancers [82, 83]. Whether related to the causative disease or to the complications of CAR-T cells, questions remain regarding the intensity of ICU management in case of CAR-T-related severe events.

## Conclusion

CAR-T cell therapies are developing rapidly with an increasingly wide range of indications for haematological malignancies, as well as potential for solid cancers and autoimmune diseases. They have demonstrated satisfying response rates and improved survival rates in patients with refractory or relapsed high-grade B cell NHL. The main CAR-T-specific toxicities are CRS, ICANS, and IEC-HS, while the main non-specific complications are infections. The most severe cases may require admission to the ICU for early management. However, the ICU admission rate, the need for organ support, and mortality tend to decline over the years.

## Data Availability

The literature review’s data are available on analysed articles from the PubMed database.

## Abbreviations

AKI: Acute kidney injury
ALL: Acute lymphoblastic leukaemia
ASTCT: American Society for Transplantation and Cellular Therapy
BCMA: B cell maturation antigen
CAR-T cell: Chimeric antigen receptor T cells
CARTOX: CAR-T cell therapy-associated Toxicity
CRS: Cytokine release syndrome
DLBCL: Diffuse large B cell lymphoma
EBMT: European Society for Blood and Marrow Transplantation
EHA: European Haematology Association
HLH: Haemophagocytic lymphohistiocytosis syndrome
ICANS: Immune effector Cells Associated Neurotoxicity Syndromes
ICU: Intensive care unit
IEC-HS: Haemophagocytic lymphohistiocytosis-like hyperinflammatory syndrome associated with immune effector cells
IL: Interleukin
JACIE: Joint Accreditation Committee ISTC EBMT
KDIGO: Kidney Disease Improving Global Outcomes
MM: Multiple myeloma
MRI: Magnetic resonance imaging
NHL: Non-Hodgkin lymphoma
PD-1: Programmed cell death-1
PDL-1: Programmed cell death ligand 1
SOFA: Sepsis-related Organ Failure Assessment
TLS: Tumour lysis syndrome

## References

[1] Hinrichs CS, Rosenberg SA (2014). Exploiting the curative potential of adoptive T-cell therapy for cancer. Immunol Rev.

[2] Eshhar Z (2008). The T-body approach: redirecting T cells with antibody specificity. Handb Exp Pharmacol.

[3] Mehrabadi AL, Ranjbar R, Farzanehpour M (2022). Therapeutic potential of CAR T cell in malignancies: a scoping review. Biomed Pharmacother.

[4] Shengnan Y, Anping L, Qian L (2017). Chimeric antigen receptor T cells: a novel therapy for solid tumors. J Hematol Oncol.

[5] Maude SL, Frey N, Shaw PA (2014). Chimeric antigen receptor T cells for sustained remissions in leukemia. N Engl J Med.

[6] Schuster SJ, Svoboda J, Chong EA (2017). Chimeric antigen receptor T cells in refractory B-cell lymphomas. N Engl J Med.

[7] Munshi NC, Anderson LD, Shah N (2021). Idecabtagene vicleucel in relapsed and refractory multiple myeloma. N Engl J Med.

[8] Schuster SJ, Bishop MR, Tam CS (2019). Tisagenlecleucel in adult relapsed or refractory diffuse large B-cell lymphoma. N Engl J Med.

[9] Neelapu SS, Locke FL, Bartlett NL (2017). Axicabtagene ciloleucel CAR T-cell therapy in refractory large B-cell lymphoma. N Engl J Med.

[10] Abramson JS, Palomba ML, Gordon LI (2020). Lisocabtagene maraleucel for patients with relapsed or refractory large B-cell lymphomas (TRANSCEND NHL 001): a multicentre seamless design study. Lancet.

[11] Bishop MR, Dickinson M, Purtill D (2022). Second-line tisagenlecleucel or standard care in aggressive B-cell lymphoma. N Engl J Med.

[12] Locke FL, Miklos DB, Jacobson CA (2022). Axicabtagene ciloleucel as second-line therapy for large B-cell lymphoma. N Engl J Med.

[13] Kamdar M, Solomon SR, Arnason J (2022). Lisocabtagene maraleucel versus standard of care with salvage chemotherapy followed by autologous stem cell transplantation as second-line treatment in patients with relapsed or refractory large B-cell lymphoma (TRANSFORM): results from an interim analysis of an open-label, randomised, phase 3 trial. Lancet.

[14] Houot R, Bachy E, Cartron G (2023). Axicabtagene ciloleucel as second-line therapy in large B cell lymphoma ineligible for autologous stem cell transplantation: a phase 2 trial. Nat Med.

[15] Sehgal A, Hoda D, Riedell PA (2022). Lisocabtagene maraleucel as second-line therapy in adults with relapsed or refractory large B-cell lymphoma who were not intended for haematopoietic stem cell transplantation (PILOT): an open-label, phase 2 study. Lancet Oncol.

[16] Wang M, Munoz J, Goy A (2020). KTE-X19 CAR T-cell therapy in relapsed or refractory mantle-cell lymphoma. N Engl J Med.

[17] Fowler NH, Dickinson M, Dreyling M (2022). Tisagenlecleucel in adult relapsed or refractory follicular lymphoma: the phase 2 ELARA trial. Nat Med.

[18] Jacobson CA, Chavez JC, Sehgal AR (2022). Axicabtagene ciloleucel in relapsed or refractory indolent non-Hodgkin lymphoma (ZUMA-5): a single-arm, multicentre, phase 2 trial. Lancet Oncol.

[19] Shah BD, Ghobadi A, Oluwole OO (2021). KTE-X19 for relapsed or refractory adult B-cell acute lymphoblastic leukaemia: phase 2 results of the single-arm, open-label, multicentre ZUMA-3 study. Lancet.

[20] Berdeja JG, Madduri D, Usmani SZ (2021). Ciltacabtagene autoleucel, a B-cell maturation antigen-directed chimeric antigen receptor T-cell therapy in patients with relapsed or refractory multiple myeloma (CARTITUDE-1): a phase 1b/2 open-label study. Lancet.

[21] Usmani S, Patel K, Hari P (2022). KarMMa-2 cohort 2a: efficacy and safety of idecabtagene vicleucel in clinical high-risk multiple myeloma patients with early relapse after frontline autologous stem cell transplantation. Blood.

[22] Rodriguez-Otero P, Ailawadhi S, Arnulf B (2023). Ide-Cel or standard regimens in relapsed and refractory multiple myeloma. New Engl J Med.

[23] Westin JR, Kersten MJ, Salles G (2021). Efficacy and safety of CD19-directed CAR-T cell therapies in patients with relapsed/refractory aggressive B-cell lymphomas: observations from the JULIET, ZUMA-1, and TRANSCEND trials. Am J Hematol.

[24] Mohty M, Dulery R, Gauthier J (2020). CAR T-cell therapy for the management of refractory/relapsed high-grade B-cell lymphoma: a practical overview. Bone Marrow Transplant.

[25] Hayden PJ, Roddie C, Bader P (2022). Management of adults and children receiving CAR T-cell therapy: 2021 best practice recommendations of the European Society for Blood and Marrow Transplantation (EBMT) and the Joint Accreditation Committee of ISCT and EBMT (JACIE) and the European Haematology Association (EHA). Ann Oncol.

[26] Zhang Q, Zu C, Jing R (2023). Incidence, clinical characteristics and prognosis of tumor lysis syndrome following B-cell maturation antigen-targeted chimeric antigen receptor-T cell therapy in relapsed/refractory multiple myeloma. Front Immunol.

[27] Howard SC, Jones DP, Pui CH (2011). The tumor lysis syndrome. N Engl J Med.

[28] Kellum JA, Lameire N, KDIGO AKI Guideline Work Group (2013). Diagnosis, evaluation, and management of acute kidney injury: a KDIGO summary (part 1). Crit Care.

[29] Kampouri E, Little JS, Rejeski K, et al. Infections after Chimeric Antigen Receptor (CAR)-T-Cell Therapy for Hematologic Malignancies. *Transpl Infect Dis.* 2023; e14157. 10.1111/tid.14157 37787373

[30] Hill JA, Krantz EM, Hay KA (2019). Durable preservation of antiviral antibodies after CD19-directed chimeric antigen receptor T-cell immunotherapy. Blood Adv.

[31] Wang D, Mao X, Que Y (2021). Viral infection/reactivation during long-term follow-up in multiple myeloma patients with anti-BCMA CAR therapy. Blood Cancer J.

[32] Hill JA, Li D, Hay KA (2018). Infectious complications of CD19-targeted chimeric antigen receptor-modified T-cell immunotherapy. Blood.

[33] Lee DW, Santomasso BD, Locke FL (2019). ASTCT consensus grading for cytokine release syndrome and neurologic toxicity associated with immune effector cells. Biol Blood Marrow Transplant.

[34] Jin Z, Xiang R, Qing K (2018). The severe cytokine release syndrome in phase I trials of CD19-CAR-T cell therapy: a systematic review. Ann Hematol.

[35] Frey N, Porter D (2019). Cytokine release syndrome with chimeric antigen receptor T cell therapy. Biol Blood Marrow Transplant.

[36] Topp MS, Gökbuget N, Stein AS (2015). Safety and activity of blinatumomab for adult patients with relapsed or refractory B-precursor acute lymphoblastic leukaemia: a multicentre, single-arm, phase 2 study. Lancet Oncol.

[37] Abboud R, Keller J, Slade M (2016). Severe cytokine-release syndrome after T cell-replete peripheral blood haploidentical donor transplantation is associated with poor survival and anti-IL-6 therapy is safe and well tolerated. Biol Blood Marrow Transplant.

[38] Norelli M, Camisa B, Barbiera G (2018). Monocyte-derived IL-1 and IL-6 are differentially required for cytokine-release syndrome and neurotoxicity due to CAR T cells. Nat Med.

[39] Rubin DB, Danish HH, Ali AB (2019). Neurological toxicities associated with chimeric antigen receptor T-cell therapy. Brain.

[40] Cordeiro A, Bezerra ED, Hirayama AV (2020). Late events after treatment with CD19-targeted chimeric antigen receptor modified T cells. Biol Blood Marrow Transplant.

[41] Neelapu SS, Tummala S, Kebriaei P (2018). Chimeric antigen receptor T-cell therapy: assessment and management of toxicities. Nat Rev Clin Oncol.

[42] Fardet L, Galicier L, Lambotte O (2014). Development and validation of the HScore, a score for the diagnosis of reactive hemophagocytic syndrome. Arthritis Rheumatol.

[43] Ramos-Casals M, Brito-Zerón P, López-Guillermo A (2014). Adult haemophagocytic syndrome. Lancet.

[44] Hines M, Knight T, McNerney K (2023). Immune effector cell-associated hemophagocytic lymphohistiocytosis-like syndrome. Transplant Cell Ther.

[45] Mehta P, Cron RQ, Hartwell J (2020). Silencing the cytokine storm: the use of intravenous anakinra in haemophagocytic lymphohistiocytosis or macrophage activation syndrome. Lancet Rheumatol.

[46] Abedin S, McKenna E, Chhabra S (2019). Efficacy, toxicity, and infectious complications in ruxolitinib-treated patients with corticosteroid-refractory graft-versus-host disease after hematopoietic cell transplantation. Biol Blood Marrow Transplant.

[47] Li S, Wang X, Yuan Z (2021). Eradication of T-ALL cells by CD7-targeted universal CAR-T cells and initial test of ruxolitinib-based CRS management. Clin Cancer Res.

[48] Shah N, Johnson B, Fenske T (2020). Intrathecal chemotherapy for management of steroid-refractory CAR T-cell-associated neurotoxicity syndrome. Blood Adv.

[49] Shah NN, Highfill SL, Shalabi H (2020). CD4/CD8 T-cell selection affects chimeric antigen receptor (CAR) T-cell potency and toxicity: updated results from a phase I anti-CD22 CAR T-cell trial. J Clin Oncol.

[50] Ganatra S, Redd R, Hayek SS (2020). Chimeric antigen receptor T-cell therapy-associated cardiomyopathy in patients with refractory or relapsed non-Hodgkin lymphoma. Circulation.

[51] Gutierrez C, Neilan TG, Grover NS (2023). How I approach optimization of patients at risk of cardiac and pulmonary complications after CAR T-cell therapy. Blood.

[52] Mahmood SS, Riedell PA, Feldman S (2023). Biomarkers and cardiovascular outcomes in chimeric antigen receptor T-cell therapy recipients. Eur Heart J.

[53] Wang Y, Zhang K, Suo X (2024). B-cell maturation antigen chimeric antigen receptor-T therapy alleviated heart failure in patients with multiple myeloma. ESC Heart Fail.

[54] Alvi RM, Frigault MJ, Fradley MG (2019). Cardiovascular events among adults treated with chimeric antigen receptor T-cells (CAR-T). J Am Coll Cardiol.

[55] Rousseau A and Zafrani L. Acute Kidney Injury after CAR-T Cell Infusion. *Bulletin Du Cancer*. 2022. 10.1016/j.bulcan.2022.08.014 36220698

[56] Kanduri SR, Cheungpasitporn W, Thongprayoon C (2021). Systematic review of risk factors and incidence of acute kidney injury among patients treated with CAR-T cell therapies. Kidney Int Rep.

[57] Lahoti A, Kantarjian H, Salahudeen AK (2010). Predictors and outcome of acute kidney injury in patients with acute myelogenous leukemia or high-risk myelodysplastic syndrome. Cancer.

[58] Brudno JN, Kochenderfer JN (2016). Toxicities of chimeric antigen receptor T cells: recognition and management. Blood.

[59] Fried S, Avigdor A, Bielorai B (2019). Early and late hematologic toxicity following CD19 CAR-T cells. Bone Marrow Transplant.

[60] Rejeski K, Perez A, Sesques P (2021). CAR-HEMATOTOX: a model for CAR T-cell-related hematologic toxicity in relapsed/refractory large B-cell lymphoma. Blood.

[61] Galli E, Allain V, Di Blasi R (2020). G-CSF does not worsen toxicities and efficacy of CAR-T cells in refractory/relapsed B-cell lymphoma. Bone Marrow Transplant.

[62] Lemoine J, Bachy E, Cartron G (2022). Non-relapse mortality after CD19 CAR T-cell therapy for diffuse large B-cell lymphoma (DLBCL): a Lysa study from the descar-T registry. Blood Adv.

[63] Le Cacheux C, Couturier A, Sortais C (2024). Features and outcomes of patients admitted to the ICU for chimeric antigen receptor T cell-related toxicity: a French multicentre cohort. Ann Intensive Care.

[64] Gutierrez C, Brown ART, Herr MM (2020). The chimeric antigen receptor-intensive care unit (CAR-ICU) initiative: surveying intensive care unit practices in the management of CAR T-cell associated toxicities. J Crit Care.

[65] Gutierrez C, Brown ART, May HP (2022). Critically ill patients treated for chimeric antigen receptor-related toxicity: a multicenter study. Crit Care Med.

[66] Azoulay E, Castro P, Maamar A (2021). Outcomes in patients treated with chimeric antigen receptor T-cell therapy who were admitted to intensive care (CARTTAS): an international, multicentre, observational cohort study. Lancet Haematol.

[67] Valade S, Darmon M, Zafrani L (2022). The use of ICU resources in CAR-T cell recipients: a hospital-wide study. Ann Intensive Care.

[68] Shimabukuro-Vornhagen A, Böll B, Schellongowski P (2022). Critical care management of chimeric antigen receptor T-cell therapy recipients. CA Cancer J Clin.

[69] Ramos CA, Grover NS, Beaven AW (2020). Anti-CD30 CAR-T cell therapy in relapsed and refractory Hodgkin lymphoma. J Clin Oncol.

[70] Chen BJ, Chapuy B, Ouyang J (2013). PD-L1 expression is characteristic of a subset of aggressive B-cell lymphomas and virus-associated malignancies. Clin Cancer Res.

[71] John LB, Devaud C, Duong CPM (2013). Anti-PD-1 antibody therapy potently enhances the eradication of established tumors by gene-modified T cells. Clin Cancer Res.

[72] Jacobson CA, Locke FL, Miklos DB (2019). End of phase 1 results from Zuma-6: axicabtagene ciloleucel (Axi-Cel) in combination with atezolizumab for the treatment of patients with refractory diffuse large B cell lymphoma. Biol Bone Marrow Transplant.

[73] Neelapu SS, Nath R, Munoz J (2021). ALPHA study: ALLO-501 produced deep and durable responses in patients with relapsed/refractory non-Hodgkin’s lymphoma comparable to autologous CAR T. Blood.

[74] Mailankody S, Matous JV, Chhabra S (2023). Allogeneic BCMA-targeting CAR T cells in relapsed/refractory multiple myeloma: phase 1 UNIVERSAL trial interim results. Nat Med.

[75] Schultz LM, Ramakrishna S, Baskar R (2022). Long-term follow-up of CD19/22 CAR therapy in children and young adults with B-ALL reveals efficacy, tolerability and high survival rates when coupled with hematopoietic stem cell transplantation. Blood.

[76] Ardeshna KM, Marzolini MAV, Norman J (2019). Phase A/2 study of AUTO3 the first bicistronic chimeric antigen receptor (CAR) targeting CD19 and CD22 folowed by an anti-PD1 in patients with relapsed/refractory (r/r) diffuse large B cell lymphoma (DLBCL): results of cohort 1 and 2 of the Alexander study. Blood.

[77] Barba P, Kwon M, Briones J (2022). YTB323 (rapcabtagene autoleucel) demonstrates durable efficacy and a manageable safety profile in patients with relapsed/refractory diffuse large B-cell lymphoma: phase I study update. Blood.

[78] Strati P, Ahmed S, Furqan F (2021). Prognostic impact of corticosteroids on efficacy of chimeric antigen receptor T-cell therapy in large B-cell lymphoma. Blood.

[79] Maus MV, Alexander S, Bishop MR (2020). Society for immunotherapy of cancer (SITC) clinical practice guideline on immune effector cell-related adverse events. J Immunother Cancer.

[80] Labanieh L, Mackall CL (2023). CAR immune cells: design principles, resistance and the next generation. Nature.

[81] Brown A, Jindani I, Melancon J (2020). ICU resource use in critically iii patients following chimeric antigen receptor T-cell therapy. Am J Respir Crit Care Med.

[82] Liu Z, Zhou J, Yang X (2023). Safety and antitumor activity of GD2-specific 4SCAR-T cells in patients with glioblastoma. Mol Cancer.

[83] Taubmann J, Müller F, Yalcin Mutlu M (2023). CD19 CAR-T cell treatment: unraveling the role of B cells in systemic lupus erythematosus. Arthritis Rheumatol.

